# Results of Kidney Transplantation from Expanded Criteria Donors: A Single-Center Experience

**Published:** 2018-02-01

**Authors:** B. Palkoci, M. Vojtko, J. Fialová, D. Osinová, M. Lajčiaková

**Affiliations:** 1 *Surgery Clinic and Transplant Center, University Hospital Martin and Jessenius Faculty of Medicine, Comenius University, Martin, Slovakia*; 2 *Department of Anaesthesiology and Intensive Medicine, University Hospital, Martin and Jessenius Faculty of Medicine, Comenius University, Slovakia*

**Keywords:** Organ donor, Transplant donor, Donor, tissue, Cadaver, Brain Death, Donor after brain death, Extended criteria donors, Kidney transplantation

## Abstract

**Background:**

Collection of kidneys from extended criteria donors (ECD) with diagnosed brain-death forms a part of the collection program that increases the number of transplantations.

**Objective:**

To compare the results of ECD with those of standard criteria donors (SCD).

**Methods:**

In a retrospective analysis in a group of 156 kidney donors, we identified ECD donors. We detected the basic parameters of the donors before kidney collection, and then evaluated the function of the graft, the survival of the graft, and the survival of the patients after 1, 3, and 5 years of transplantation. The results were then compared with the function of the graft from those of SCD donors.

**Results:**

The ECD donors were significantly (p<0.001) older than the SCD donors. They had a higher body mass index (p=0.006) and prevalence of hypertension (p<0.001) and diabetes mellitus (p=0.004) compared to SCD donors. The graft function within the first 6 months and the survival of recipients in the first year of transplantation were significantly worse in ECD than in SCD groups (p=0.01, and 0.023, respectively). No difference in the graft survival was observed between the two groups.

**Conclusion:**

The long-term function of the graft and survival of patients and grafts in recipients of kidneys from ECD donors are comparable to SCD donors. Exploitation of the given organs for transplantation is important due to the constantly increasing demand versus limited offer of organs.

## INTRODUCTION

Kidney transplantation is the treatment of choice for patients with end-stage renal failure. The quality of life and the survival of patients are significantly higher in patients after kidney transplantation compared to those in the waiting list [1]. Three-quarters of patients after kidney transplantation are able to re-enter the working process; approximately, one of 50 women in reproductive age with transplanted kidney becomes pregnant. The lack of organs from dead donors and the constantly increasing number of patients in the waiting list results in certain compensating and alternative strategies. Collection of kidneys from extended criteria donors (ECD) with diagnosed brain-death is included in the collection program and increases the number of transplantations [2].

The Organ Procurement and Transplantation Network (OPTN) instituted a formalized definition of marginal kidneys in 2002 with the advent of ECD. ECD donors are normally aged 60 years or older, or over 50 years with at least two of the following conditions: having history of hypertension with a serum creatinine level of >1.5 mg/dL, or dying of cerebrovascular accident [3]. ECD kidneys are those taken either from a brain-dead donor ≥60 years of age, or a donor aged 50–59 years with at least two of the following features: having history of hypertension, terminal serum creatinine level of >1.5 mg/dL (133 mmol/L), or dying of cerebrovascular causes [4]. These criteria for definition of ECD are based on the presence of variables that increase the risk for graft failure by 70% (relative HR of 1.70) *vs* the standard criteria donor (SCD). Kidney transplants coming from donation after cardiac death (DCD) are not included in this definition. SCD is defined as a donor who fails to meet the criteria for DCD or ECD [5].

Based on many large retrospective database analyses, kidneys transplanted from ECDs have higher delayed graft function (DGF) rates, more acute rejection episodes, and decreased long-term graft function. An ECD kidney transplant recipient has a projected average added-life-years of 5.1 years *vs* 10 years for a kidney recipient from an SCD [6, 7]. Despite these inferior results, these transplants have definitely survival advantages over dialysis patients remaining on the transplant waiting list [7]. We conducted this study to compare the results of ECD with those of SCD donors.

## MATERIALS AND METHODS

In 156 kidney donors in the Transplant Center Martin, we retrospectively identified ECD donors according to the OPTN criteria. We recorded the donor’s age, sex, cause of death, comorbidities (diabetes mellitus, arterial hypertension), and laboratory parameters before collection of the transplant (the estimated glomerular filtration [eGFR] according to CKD-EPI, creatinine level, presence of proteinuria, and serum sodium, potassium and chlorides). We compared the parameters in the ECD with SCD donors. Furthermore, in each group of recipients, we recorded the function of the graft by eGFR in after 1, 3, 6, 12, 36, and 60 months of transplantation. We also assessed the type of the induction used (basiliximab, antithymocyte globulin, or no induction) and immunosuppression (tacrolimus, cyclosporine A), and the duration of cold ischemia. We evaluated the onset of the function of the graft (primary or delayed-onset of the function, requiring dialysis in the post-transplantation period), and occurrence of acute rejection within 12 months of transplantation. We also monitored surgical complications within 30 days of transplantation (bleeding, stricture of ureter, or lymphocele). The given parameters were compared between the recipients of ECD and SCD grafts. Finally, we compared the graft and recipient survivals between of the recipients of ECD and SCD grafts 12 and 60 months of transplantation.

Ethics

All procedures performed in studies involving human participants were approved under the ethical standards of the institutional and/or national research committee and under the 1964 Helsinki Declaration, as amended, or the comparable ethical standards.

Statistical Analysis

Medcalc^®^ *ver* 13.1.2 was used for statistical data analysis. Student’s t test, χ^2^ test and Kaplan-Meier survival analysis were used. A p value <0.05 was considered statistically significant.

## RESULTS

The studied donors (n=156) had a mean±SD age of 46±16 years. There were 50 ECD and 107 SCD donors (Table 1). ECD donors had a significantly higher age and body mass index (BMI). The prevalence of hemorrhagic cerebrovascular accident as the cause of death, and diabetes mellitus was also higher in ECD than SCD donors.

**Table 1:** Characteristics of the studied groups. Values are mean±SD or percentage.

Parameter	SCD (n=107)	ECD (n=50)	p value
Age (yrs)	40±13	60±5.3	<0.001
Male	71.0%	64.4%	0.423
BMI (kg/m^2^)	24.5±2.8	26.1±4	0.006
History of alcohol abuse	25.2%	28.9%	0.637
Cause of death			
Cranio-trauma	57.9%	20.0%	<0.001
Hemorrhagic CVA	24.3%	75.6%	<0.001
Ischemic CVA	15.0%	4.4%	0.066
TU of brain	2.8%	0.0%	0.259
History of hypertension	20.6%	64.4%	<0.001
History of diabetes mellitus	2.2%	20.6%	0.004
Serum creatinine before collection (µmol/L)	93.2±26.4	88.8±23.8	0.336
eGFR before collection (mL/s)	1.41±0.65	1.23±0.33	0.080
Best adjusted GFR (mL/s)	1.89±0.88	2.06±2.96	0.589
[Na] before collection (mmol/L)	142±10	142±8.3	1.000
[K] before collection (mmol/L)	4.1±0.5	4.1±0.4	1.000
[Cl] before collection (mmol/L)	112±10	111±8.6	0.559
QP before collection (g/collection)	0.436±0.426	0.634±1.153	0.124

BMI: body mass index; CVA: cerebrovascularaccident; TU: tumor; eGFR: estimated glomerular filtration rate according to CKD-EPI formula; GF: glomerular filtration rate; [Na]: serum sodium concentration; [K]: serum potassium concentration; [Cl]: serum chloride concentration, QP: quantitative proteinuria

**Figure 1:**
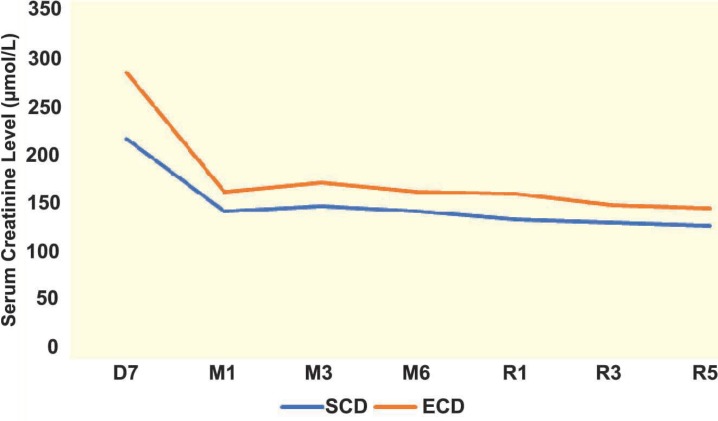
Trend of serum creatinine level (µmol/L) at various times post-kidney transplantation (recipients of organs from SCD vs ECD donors)

**Figure 2:**
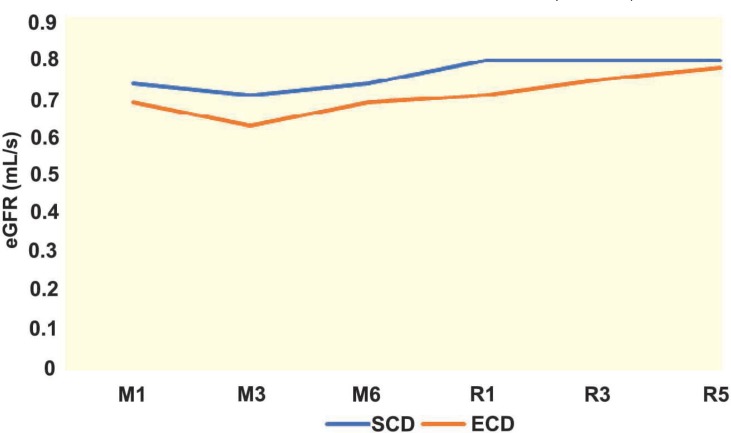
eGFR at various time post-kidney transplantation (recipients of organs from SCD vs ECD donors)M: month after transplantation; Y: year after transplantation

Characteristics of recipients of ECD and SCD grafts are shown in Table 2. The recipients of ECD grafts were significantly older than recipients of SCD. The frequency of patients who were administered antithymocyte globulin for induction was significantly higher than that in SCD. Serum creatinine level was also higher in the recipients of ECD than SCD grafts. However, the function of the graft, assessed by eGFR, was lower only during the first year after transplantation; no significant difference was observed in the function of the graft between the two groups, thereafter. The trends of serum creatinine and eGFR over time are presented in Figures 1 and 2. Receiving an ECD graft had no effect on the function of the graft; nor did it affect the rejection of the graft within the 12 months of transplantation. We did also not record higher incidence of post-operative complications.

**Table 2:** Characteristics of recipients of kidney from ECD *vs* SCD donors. Values are mean±SD or percentage

Parameter	SCD (n=107)	ECD (n=50)	p value
Age at the time of transplantation (yrs)	46.9±11.5	51.3±10.4	0.020
Male	57.4%	82.0%	0.002
Duration of cold ischemia (min)	687.2±336.5	666.5±253	0.694
Induction			
No	13.8%	28.3%	0.027
Basiliximab/daclizumab	77.7%	47.8%	<0.001
ATG	8.6%	23.9%	0.007
Tacrolimus	79.2%	84.8%	0.410
Cyclosporine A	20.8%	15.2%	0.410
Revision due to bleeding	6.2%	6.0%	0.960
Lymphocele	5.4%	0.0%	0.095
Stricture of ureter	5.4%	2.0%	0.324
Primary function of the graft	82.9%	72.0%	0.104
Delayed function of the graft	10.9%	14.0%	0.565
Acute rejection			
Within 12 months of transplantation	20.2%	18.0%	0.740
After 12 months of transplantation	27.1%	38.0%	0.155
Serum creatinine level (µmol/L)			
7^th^ day	218.0±185.0	287.0±238.0	0.041
1^st^ month	143.0±42.9	163.0±44.2	0.006
3^rd^ month	148.0±39.9	172.0±44.9	<0.001
6^th^ moth	142.9±45.5	163.0±44.9	0.009
1^st^ year	133.6±40.6	161.0±62.4	<0.001
3^rd^ year	130.7±37.4	149.0±41.0	0.005
5^th^ year	127.6±28.4	146.0±41.8	<0.001
eGFR (mL/s)			
1^st^ month	0.74±0.19	0.67±0.18	0.026
3^rd^ month	0.71±0.18	0.63±0.17	0.007
6^th^ month	0.74±0.20	0.69±0.21	0.011
1^st^ year	0.80±0.21	0.71±0.21	0.141
3^rd^ year	0.80±0.17	0.75±0.21	0.108
5^th^ year	0.80±0.19	0.78±0.22	0.547

ATG: antithymocyte globulin; eGFR: estimated glomerular filtration rate according to CKD-EPI formula

D: day after transplantation; M: month after transplantation; Y: year after transplantation

The incidence of BK virus infection diagnosed by polymerase chain reaction (PCR) was 8.4% in SCD and 8% in ECD group (p=0.933). We did not record any graft loss because of BK infection in 12 months of transplantation. Replication of cytomegalovirus (CMV) was recorded in 45.8% of recipients in SCD group and in 45.2% of recipients in ECD group (p=0.929); there was no significant difference between the mean CMV DNA copies within the monitored period between the two groups (3500 copy/mL in SCD *vs* 3800 copy/mL in ECD; p=0.976).

**Figure 3:**
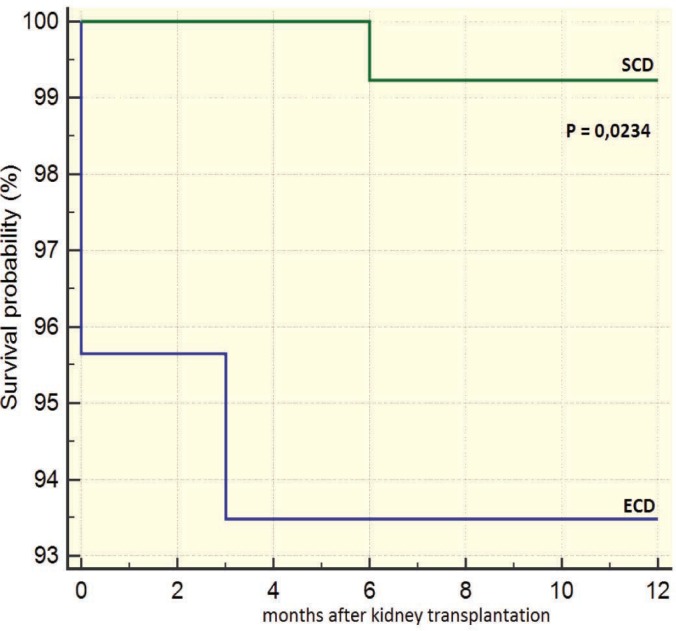
12-month survival of recipients of ECD vs SCD grafts

**Figure 4:**
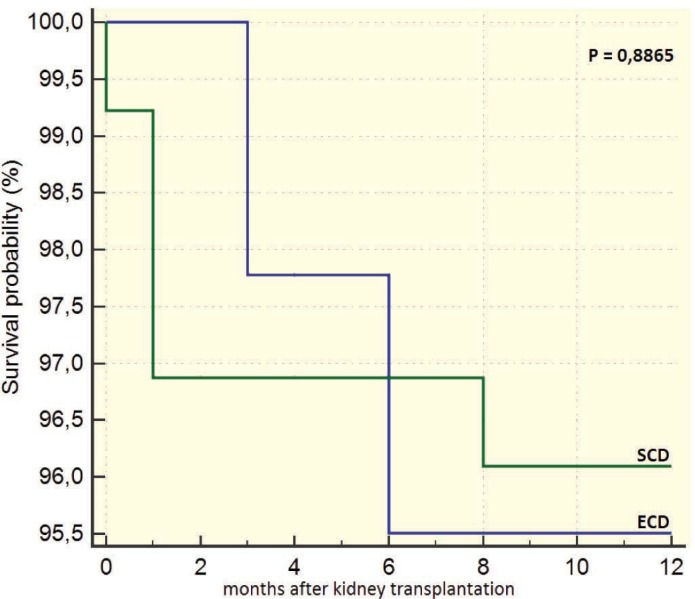
12-month survival of grafts taken ECD vs SCD donors

**Figure 5:**
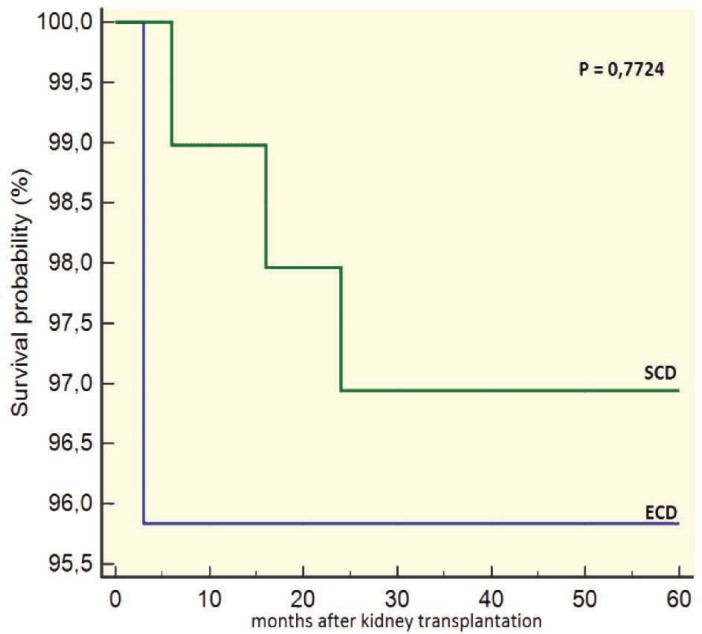
5-year survival of recipients of ECD *vs* SCD grafts

**Figure 6:**
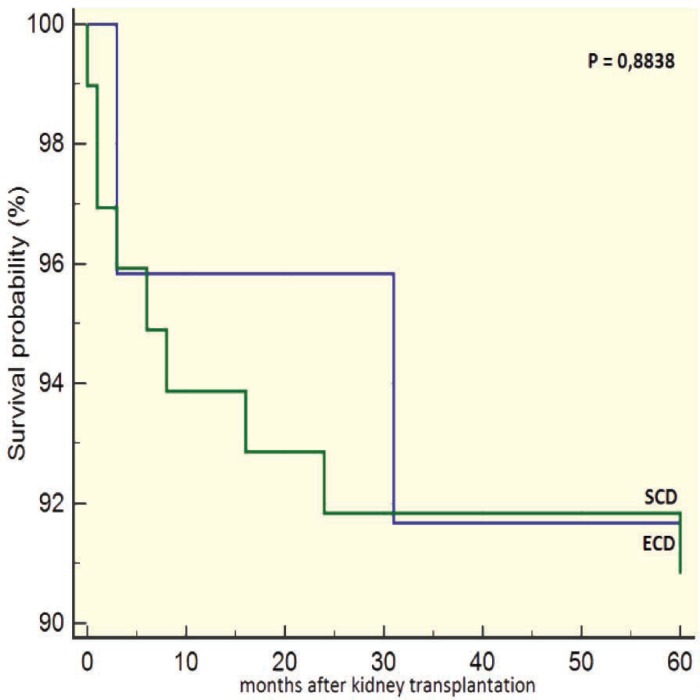
5-year survival of grafts from ECD *vs *SCD donors

Recipients of ECD grafts had a worse survival than those of SDC grafts after 12 months of transplantation (93.6% vs 99.3%, p=0.023; Fig 3). However, there was no significant difference in the death censored survival after 12 months of the grafts from ECD vs SCD donors (95.5% vs 96.1%, p=0.887; Fig 4). The survival of recipients of grafts from ECD donors five years after transplantation was 96%; it was 97% in recipients of SCD grafts (p=0.772). The death censored survival of grafts from ECD and SCD donors five years after transplantation was not significant different (91.9% vs 92%, p=0.884; Figs 5 and 6).

Using multivariate analysis, it was found that delayed graft function and acute rejection within 12 months of transplantation were independent predictors for worse graft function in the 12^th^ month after kidney transplantation (defined as serum creatinine level >110 µmol/L in men and >96 µmol/L in women) (Tables 3 and 4).

**Table 3:** Results of the logistic regression analysis

Variable	OR (95% CI)	p value
Primary function of the graft (%)	0.09 (0.04–0.23)	0.371
Delayed function of the graft (%)	0.35 (0.04–2.79)	<0.001
Acute rejection within 12 months of transplantation	33.25 (10.01–110.45)	<0.001
Induction		
No (%)	1.67 (0.67–4.16)	0.271
Basiliximab/daclizumab (%)	1.19 (0.51–2.76)	0.693
ATG (%)	0.18 (0.02–1.40)	0.102
ECD donor (%)	2.99 (0.86–10.46)	0.086
CMV replication (%)	0.39 (0.13–1.21)	0.103
BKV nephropathy (biopsy-proven)	0.06 (3.49–4.93)	0.836

ATG: antithymocyte globulin; CMV: cytomegalovirus

**Table 4:** Results of Cox’s regression analysis

Variable	HR (95% CI)	p value
Primary function of the graft (%)	0.37 (0.05–2.78)	0.335
Delayed function of the graft (%)	0.53 (0.37–0.76)	<0.001
Acute rejection within 12 months of transplantation	17.38 (6.25–48.31)	<0.001
Induction		
No (%)	1.75 (0.87–3.53)	0.114
Basiliximab/daclizumab (%)	1.15 (0.57–2.30)	0.695
ATG (%)	0.18 (0.03–1.24)	0.082
ECD donor (%)	2.55 (0.83–7.84)	0.103
CMV replication (%)	0.33 (0.10–1.17)	0.086
BKV nephropathy (biopsy-proven)	1.08 (0.73–1.60)	0.703

ATG: antithymocyte globulin; CMV: cytomegalovirus

## DISCUSSION

Lack of organs for the high demand led to increased number of kidney transplantations from ECD donors. Most of the studies confirm that the grafts from ECD donors have worse survival and function compared to SCD grafts. However, the survival of recipients of ECD grafts is obviously better than those in the waiting list. Naturally, the results of transplantations of kidneys from ECD donors are also related to the recipient’s characteristics, namely his comorbidities [8]. Several studies show that, for younger patients, it is generally worth waiting for a higher-quality kidney. For older patients, nonetheless, prolonged waiting for an SCD kidney is not in their interest [9, 10]. In our study we confirmed that the recipients of kidneys from ECD donors were significantly older than the recipients of kidneys from SCD donors. In electing the induction in our patients, we used significantly more often antithymocyte globulin for ECD kidneys. Patients transplanted with ECD kidneys are more likely to experience delayed graft function (DGF) and diminished allograft function, resulting in increased resource utilization and higher risk of graft loss [11]. However, in the induction therapy, neither increased incidence of rejection, nor delayed-onset graft function were observed in the studied kidney recipients. The goal of any immunosuppression protocol should be to achieve an adequate immunosuppression level that offers minimum risk of infection without increasing the risk of rejection. In any case, older patients and recipients of ECD kidneys are often excluded from transplant trials and, thus, the optimum induction and maintenance regimen for these group of recipients is unknown. Approaches are largely center-specific and based upon expert opinion [12, 13].

The function of the ECD graft in our group, in the first six months of transplantation, was significantly worse than the grafts from SCD donors. However, the function in both groups were comparable, thereafter. A meta-analysis in 2008 showed that ECD kidneys have worse long-term survival than the SCD kidneys. The optimum ECD kidney for donation depends on the adequate glomerular filtration rate and acceptable donor kidney histological characteristics, albeit the usefulness of biopsy is under debate [14]. A retrospective analysis also showed that renal transplantation with grafts from ECD has a significantly worse outcome with higher rates of delayed graft function and acute rejection, worse graft function, and lower graft survival [15]. The survival of the grafts from ECD and SCD donors were in our group comparable five years of transplantation. The survival of recipients of ECD grafts in the 1^st^ year after transplantation was significantly worse than that of recipients from SCD donors. We believe this would relate to the higher age of the recipients of kidneys from ECD donors (with presumably more comorbidities). A conclusion similar to our analysis was also made by Kim, *et al*, who retrospectively evaluated the results of organs from ECD donors. They found that the graft survival of ECD kidneys was comparable to that of SCD kidneys. They observed that the donor factors prior to the organ procurement have no effect on the subsequent graft failure [16]. 

In conclusion, the function of the graft and the survival of patients and grafts in recipients of kidneys from ECD donors is comparable, in long-term, to recipients of kidneys from SCD donors. Use of such organs for transplantation is important, mainly in the current situation of increasing demand with limited offer of organs [17, 18]. Appropriate selection of ECD kidney transplant recipients and close peri- and post-operative follow-up of patients are of prime importance in order to maximize the benefits associated with the increasingly widespread use of ECD kidney allografts.

## CONFLICTS OF INTEREST:

None declared.
